# Social Isolation Shortens Telomeres in African Grey Parrots (*Psittacus erithacus erithacus*)

**DOI:** 10.1371/journal.pone.0093839

**Published:** 2014-04-04

**Authors:** Denise Aydinonat, Dustin J. Penn, Steve Smith, Yoshan Moodley, Franz Hoelzl, Felix Knauer, Franz Schwarzenberger

**Affiliations:** 1 Department of Biomedical Sciences, Biochemistry, University of Veterinary Medicine, Vienna, Austria; 2 Department of Integrative Biology and Evolution, Konrad Lorenz Institute of Ethology, University of Veterinary Medicine, Vienna, Austria; 3 Department of Integrative Biology and Evolution, Research Institute of Wildlife Ecology, University of Veterinary Medicine, Vienna, Austria; University of Newcastle, United Kingdom

## Abstract

Telomeres, the caps of eukaryotic chromosomes, control chromosome stability and cellular senescence, but aging and exposure to chronic stress are suspected to cause attrition of telomere length. We investigated the effect of social isolation on telomere length in the highly social and intelligent African Grey parrot *(Psittacus erithacus erithacus)*. Our study population consisted of single-housed (n = 26) and pair-housed (n = 19) captive individuals between 0.75 to 45 years of age. Relative telomere length of erythrocyte DNA was measured by quantitative real-time PCR. We found that telomere length declined with age (p<0.001), and socially isolated parrots had significantly shorter telomeres compared to pair-housed birds (p<0.001) – even among birds of similar ages. Our findings provide the first evidence that social isolation affects telomere length, which supports the hypothesis that telomeres provide a biomarker indicating exposure to chronic stress.

## Introduction

Individual variation in ageing and health are associated with differences in telomere length (TL) [1]. Telomeres are specialized protein-DNA complexes, which provide a protective cap on the end of linear chromosomes and prevent chromosomal degradation and end-to-end fusions by ‘hiding’ free DNA ends. *In vitro* studies show that telomeres become shorter with every cell division due to the inability of DNA polymerase to fully replicate the 5′end of linear DNA (‘end-replication problem’). Once a critical TL is reached, cells enter replicative senescence and irreversibly lose their potential to divide. [2], [3] Cellular senescence provides a mechanism to eliminate cells with DNA damage and protect against cancer [4], [5], but it also impairs cell function and appears to contribute to organismal aging and mortality [1]–[10]. Numerous studies have found that short telomeres are associated with increased risk of a variety of (age-related) diseases in humans [6] and reduced lifespan in different species [7]–[10]. The causes of variation in telomere shortening (and repair) among individuals are not fully understood, though the leading hypothesis suggests they are due to differences in exposure to chronic stress and lifestyle [1], [11], [12].

Chronic exposure to stressful conditions interferes with anti-oxidative defences, which increase cellular and DNA damage (reviewed by [13], [14]), especially in the telomeric region [15]. In addition, oxidative telomere erosion has also been proposed to contribute to disrupted telomere repair mechanisms [16]. For example, telomerase, the enzyme responsible for TL elongation, is vulnerable to oxidative stress. Some studies show diminished activity of telomerase due to chronic stress [11], and though other studies found the opposite relationship [17], [18], experimental disruption of telomerase expression reduced TL and lifespan in mice and zebrafish [19], [20]. Taken together, increased oxidative damage, and inefficient repair due to negative external lifestyle factors are the leading explanations for accelerated telomere shortening. For example, in humans, it was initially discovered that mothers exposed to higher levels of chronic psychological stress (caring for chronically ill children) showed higher levels of oxidative stress together with shorter TLs than other mothers [11]. Subsequent studies in humans have also found exposure to a variety of stressors – including psychological problems (e.g., depression [21] and mood disorders [22]), smoking, obesity [23], and infectious diseases [24] – are associated with reduced TL. Furthermore, experimental tests with house mice support the hypothesis that exposure to stressful conditions cause telomere shortening [25], [26]. Some studies in birds show that longer telomeres are associated with enhanced survival [9], [27] and lifetime reproductive success [28]. Yet, the fitness consequences of TL shortening are still debated because other studies in humans found no effects on mortality [29] and some show telomere lengthening with chronological age [30], [31].

We aimed to test whether and how social isolation affects telomere length in single-housed versus pair-housed captive African Grey parrots *(Psittacus erithacus erithacus)*. Social isolation is a highly stressful situation for social species, and though it may have long-lasting consequences [32], most studies have only assessed endocrinological (especially corticosteroids) and neurochemical effects [33], [34]. African Grey parrots are long-lived birds and reach sexual maturity between 4–7 years of age, and in the wild, they roost in large flocks of up to 10,000 birds and forage in groups of up to 30 individuals [35]. They form life-long monogamous pairs, and they are rarely (if ever) socially isolated for long periods. In captivity, however, these parrots are often kept solitary, which can have a variety of detrimental effects. Psychogenic feather damaging behaviour and other self-mutilation, aggression, screaming, neo-phobias and stereotypic behaviour with the absence of medical causes and constantly recurring illnesses are often described in parrots kept in deprived environments [36]–[40]. Hand-rearing causes a variety of permanent behavioral disorders in African Grey parrots, which appear to be due to mal-imprinting on human surrogate parents [41]. Yet, since social development extends beyond the nesting period and includes interactions with other conspecifics, we expect social isolation to cause long-lasting physiological and behavioral disorders, even in parrot-reared birds. Our goals were to test whether (1) TL declines with age, (2) telomere attrition is accelerated in socially isolated birds, and (3) telomere attrition is associated with reduced longevity.

## Material and Methods

### (a) Ethical statement

No Ethics permissions were needed according to the Ethics Commission of the University of Veterinary Medicine Vienna because blood samples were not collected for our study (they were collected in routine veterinary exams requested by the owners, and all efforts were made to minimize suffering to the animals in these exams). Austrian legislation has not permitted single housing of parrots since 2005, but it is still common to encounter single-housed parrots in veterinary practice.

### (b) Animal subjects, housing and survival

Subjects were 45 captive African Grey Parrots (*Psittacus erithacus erithacus*), 21 females and 24 males of different ages (0.75–45 years) from routine check-ups in a small animal clinic in Vienna between 2011 and 2013. All individuals were bred in captivity and housing conditions were obtained by interviewing the owners. Only individuals of known age (i.e., ringed individuals or birds acquired as juveniles with known year of hatching) and living in either single- or pair-housing (a group of two birds, but not necessarily consisting of a male and female) throughout their entire lives were used for the study. Birds reared in different or unknown housing conditions were not included in the study. None of the birds in the study reproduced. African Grey parrots are very long-lived birds (the longest reliably recorded life expectancy in captivity is 49 years [42]). It was not feasible to track individual life expectancy, however, and patient records were used to track survival of the birds until the end of the study.

### (c) Relative telomere length (RTL) measurement and sexing

We obtained RTLs from erythrocyte DNA. Blood was collected from the brachial vein with a heparinized 1 ml syringe. After centrifugation and separation from plasma, 32 μl of the erythrocyte fraction was transferred to 1 ml Queens lysis buffer and stored at 4°C until analysis. DNA was extracted prior to RTL analysis using a commercial kit (DNeasy Blood&Tissue Kit, Qiagen) and RTLs were obtained with a quantitative real-time PCR method [43]–[45] adapted for our species. This (qPCR) method measures the relative amount of telomeric DNA (i.e., number of telomeric repeats versus a single copy control) rather than absolute telomere length (TL) per se. DNA from birds is known to contain interstitial telomere repeats away from chromosome ends, but relative TL measurement via qPCR has been well validated in birds and the interstitial repeats only increase noise in the estimates, rather than generating false positive results [44], [46]. We used a 72 bp sequence of GAPDH (segment of GenBank Acc. N. JN614810) as the non-variable copy number gene (non-VCN gene), which was selected for non-variability as described by Smith et al. [47]. Primer specificity was ensured by gel-electrophoresis. Forward and reverse telomeric primers were 5′-CGGTTTGTTTGGGTTTGGGTTTGGGTTTGGGTTTGGGTT-3′ (tel 1b) and 5′-GGCTTGCCTTACCCTTACCCTTACCCTTACCCTTACCCT-3′ (tel 2b), respectively, and forward and reverse primers for the non-VCN gene (GAPDH) were 5′-CAGGCTTGTGTCCTGTTTTG-3′ (GAPDH72r) and 5′GAGACACCCTCCTCAGCAAG-3′ (GADPH72f), respectively. Telomere and non-VCN gene PCRs were carried out in separate runs using 40 ng DNA per reaction in a Rotorgene Q (Qiagen, Germany) instrument. Primer pairs (Tel1b/Tel2b or GAPDH72r/GAPDH72f) were used in a concentration of 400 nM each, in a final volume of 20 μl containing 10 μl of SensiMix SYBR No-ROX-MasterMix (Bioline). PCR conditions for the telomere primers were 10 min at 95°C followed by 35 cycles of 10 sec at 95°C, 20 sec at 56°C and 20 sec at 72°C. For GADPH PCR conditions were 10 min at 95°C followed by 40 cycles of 10 sec at 95°C, 20 sec at 60°C and 20 sec at 72°C. PCR efficiencies and Ct-values (cycle threshold) were computed directly, without the use of standard curves with LinRegPCR software (2012.0) [48]. LinRegPCR uses linear regression to estimate individual sample reaction efficiencies of non-baseline-corrected raw qPCR data and these efficiency values were then directly incorporated into the calculation of RTL. Mean qPCR efficiencies were 99.4% and 97.8% for the telomere and non-VCN gene reactions (correlation coefficient r^2^≥0.999). To be able to compare RTL among plates, all telomere to non-VCN ratios were normalised to one individual (reference standard sample, RTL = 1), which was included in every qPCR run. Using the mean PCR efficiency per amplicon (E) and the Ct-value per sample, the RTL was calculated using the following formula [49], [50]:
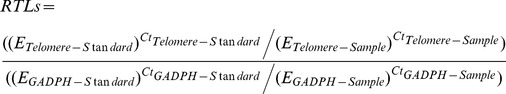



All samples were run in triplicate to confirm accuracy and reproducibility. The co-efficient of variation among replicates (intra-assay variance) for Ct-values of GADPH assays was 0.3% and 0.87% for the telomere assays. We chose a generally randomized sample setup. To examine possible run-to-run variations, we re-measured three samples (including the reference standard sample), again in triplicates, across every run. We then determined the coefficient of variation for RTL. The inter-assay mean co-efficient of variation of these controls was 4.9%. A no-template control (NTC, in triplicate) was also included in each run. For the non-VCN assay, amplification of the NTC above threshold levels was not detected and for the telomere assay no amplification was detected until at least 18 cycles after the weakest signal of a sample (clear of the allowable range according to accepted qPCR guidelines [51]). A final melt step was included for each run to check for target specificity via unimodal melt dissociation peaks. The temperature ramping was set from 65°C to 95°C in 1°C steps. For all assays, only the expected peaks for telomere and non-VCN genes were observed. A pipetting robot (Qiagility, Qiagen, Germany) was used for all assay runs to minimize pipetting errors and ensure consistency in reactions. African Grey parrots are sexually monomorphic; therefore, sex was determined with a standard PCR protocol (Primer Pair 2550F/2718R [52]).

### (d) Statistical analysis

To test the effects of age, housing and sex on RTL we applied a generalized additive model (GAM) with RTL as the dependent variable, housing (single-or pair-housing) and sex as fixed factors and age as a spline. To evaluate and compare different models, we used the Akaike Information Criterion (AICc), corrected for small sample sizes. There was not one best model and therefore we used model averaging and multimodel inference [53]. Model assumptions were checked by visual inspection of the residuals. The Cox proportional hazard model was applied to assess the effect of RTL on mortality. RTL and age were used as covariates. In this model, we included the full data set and did not distinguish between pair-housed and single-housed birds. Individuals that were still alive at the end of the study period formed the censor group. Statistical analysis was performed using the software R 2.12.2×64 (R Development Core Team, 2011). GAM estimation and model averaging was conducted with the package mgcv [54] and MuMIn [55], respectively. Cox proportional hazard model was conducted with the software SPSS (IBM SPSS Statistics 20).

## Results

**Figure 1:**
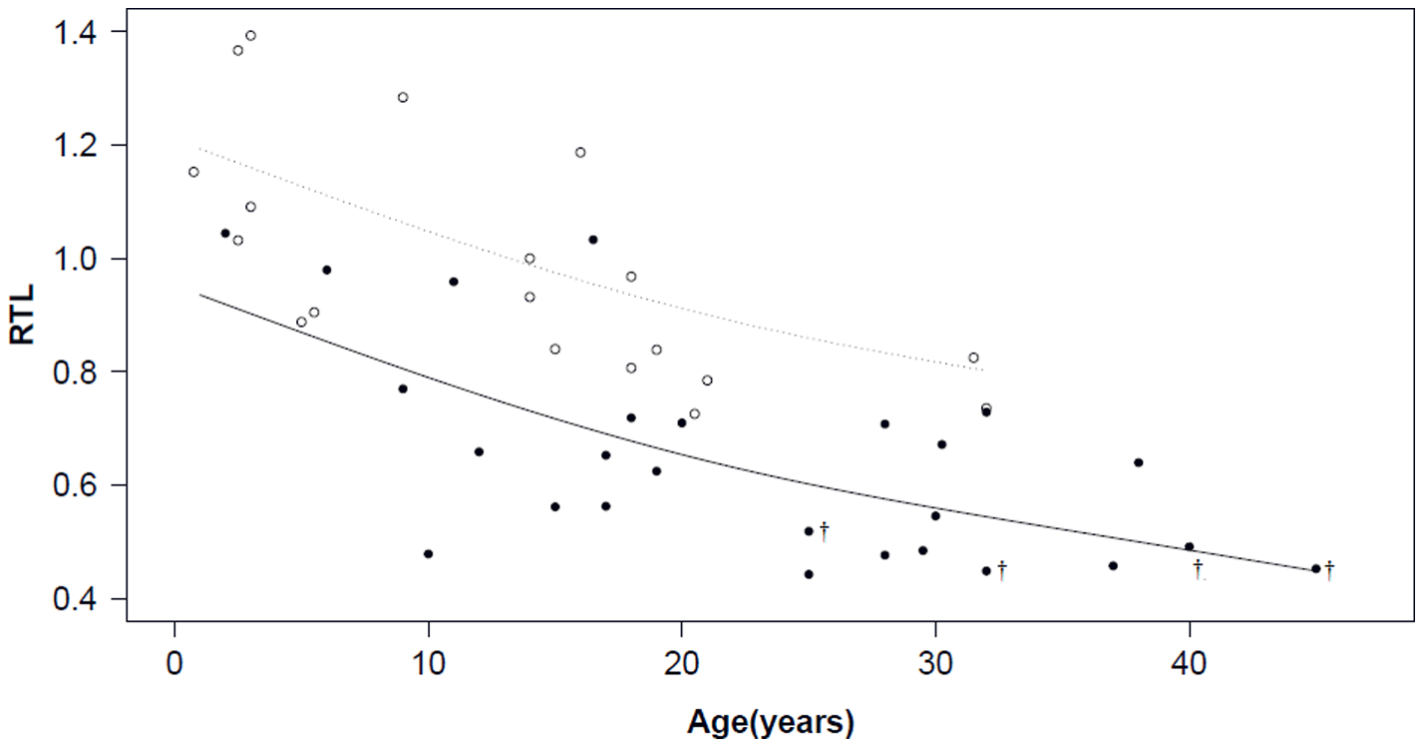
Relative telomere length (TL/non-NVC) and age for single-housed birds (filled circles) and pair-housed birds (open circles); Deviance explained 70.6%; † individuals that died during study period. The predicted RTLs are shown with a black line for single-housed birds and with a dashed line for pair-housed birds.

Overall, relative telomere length (RTL) declined with age (n = 45; GAM, p<0.001), and single-housed birds had shorter telomeres compared to the group of pair-housed individuals (single-housed: n = 26; pair-housed: n = 19; GAM, p<0.001). Figure 1 shows the negative correlation between age and RTL for both single- and pair-housed birds and the differences in their relative TL, even among birds of the same age. There was no significant relationship between sex and RTL. Estimated effect sizes, relative variable importance, and p-values are shown in Table 1. Of these 45 individuals, a small number of individuals (n = 4 or 9%) died (non-traumatically) during the study period. Using the Cox proportional hazard model we found a positive but non-significant effect of age on mortality (β = 0.109; SE = 0.72; p = 0.129) and a negative, but also non-significant effect of RTL on mortality (β = −14.918; SE = 10.331; p = 0.149). (Data for this study are available upon request from the corresponding author)

**Table 1:** Estimated effect size, adjusted standard error (SE), z-value and relative variable importance (RVI) estimated by a generalized additive model to determine the effects of age (as spline), housing and sex on RTL (n = 45).

	Effect size	Adjusted SE	z-value	P-value	RVI
Intercept	0.685	0.038	17.805	<0.001	
Age			14.11^1^	<0.001^2^	1
Sex	0.053^3^	0.044	1.155	0.248	0.37
Housing	0.227^4^	0.049	4.494	<0.001	1

1 F-value from full model.

2 taken from the full model.

3 effect size by males over females.

4 effect size by pair-housing over single-housing.

## Discussion

We found a decline in RTL with age, as previously reported in mammals and other avian species [3], [28], and moreover, we found that single-housed individuals had shorter RTL than pair-housed birds. Based on our model, the RTLs of single-housed birds at nine years of age were comparable to pair-housed birds 23 years older than themselves. Our model controlled for the difference in the age distribution of the pair- and single-housed birds. Even when we restricted our analysis to individuals with comparable ages (between 0.75–32 years), our results did not change qualitatively. Although our findings reveal that social isolation impacts telomere length, we found no significant difference in the rate of decline between single-housed versus pair-housed birds. This result implies that rather than accelerating the rate of TL attrition with age, shorter RTL of isolated birds have developed in early life (before the age when our sample collection started) and a lack of telomere repair in subsequent life (i.e., carry-over ontogenetic effects). Thus, there might be a critical period in early life that explains our results. However, we had only few samples for animals less than one year old and no data about the early life of these birds are available, and therefore further studies testing birds less than five years of age (before reaching sexual maturity) are necessary. Exposure to early-life stress is often proposed to explain reductions in TL later in life [56]. As the decline in RTL with age between the two groups occurred in parallel, it is possible that isolated birds may have habituated to their aberrant social situation or adopted their owners as substitute conspecifics. Since TL attrition has been found to provide a predictor of mortality in other species [8], [57], [10], we tested whether short telomeres had negative fitness consequences. Few subjects died during the study period, and though we found a suggestive trend, we found no significant effect of RTL on adult survival. In summary, our findings support the hypothesis that ageing and exposure to social isolation both reduce telomere length, and though we found no significant effects of telomere attrition on survival, a larger study and preferably using longitudinal sampling is needed to adequately test the consequences of TL attrition on longevity.

To our knowledge, these findings are novel for non-human species, yet they are consistent with human studies, which have found that chronic social deprivation (i.e., foster children reared in institutional care [58] and adults experiencing low social support and loneliness [59]) are associated with shorter TL later in life. These human studies measured the effects of social deprivation rather than complete isolation from conspecifics, as in our study, though the underlying mechanisms leading to shorter TL might be similar. Chronic stress from social isolation or deprivation could affect TL through increased oxidative stress, higher cellular turnover, disturbed telomere maintenance and less efficient repair [13], [14]. Regardless of the underlying mechanisms, our findings suggest that TL may be used as a biomarker for identifying individuals suffering from social stress. TL could also be a useful tool for studying animals in wild populations that are chronically stressed by anthropogenic disturbance and environmental changes [60]. Traditionally, exposure to stress has been assessed predominantly by measuring corticosteroids; however, the high individual variation in endocrine responses during chronic stress has raised doubts about the predictive value of this method [61]. Contrary to the widespread assumption that elevated corticosteroids provide a universal marker of chronic stress, experimental evidence suggest the possibility of attenuated baseline levels in chronically stressed animals [62]. Further experimental or longitudinal studies are needed to test whether and how TL can provide an additional biomarker for identifying exposure to chronic social stress. TL should be more useful as part of an overall composite index, included in addition to other biomarkers of oxidative stress and aging. It is unknown whether or not the negative effects of chronic stress exposure on telomere attrition might be slowed down or reversed by transferring animals from a solitary to a social environment, though our findings suggest interesting experimental tests for future studies.
